# Willingness to donate biosamples to a national biobank in Israel: a population-based cross-sectional survey

**DOI:** 10.1186/s13584-026-00762-3

**Published:** 2026-05-06

**Authors:** Adelina Ovcharenko, Yehudit Cohen, Shimon A. Reisner

**Affiliations:** MIDGAM, Israel National Biobank for Research, 39 Yirmiyahu Street, Jerusalem, 9446724 Israel

**Keywords:** Biobank, Biosamples, Willingness to donate, Barriers

## Abstract

**Background:**

Israel is advancing large genetic and longitudinal studies in personalized medicine through national digital health initiatives. MIDGAM, the Israeli national biobank established in 2014, provides the core biosample and data infrastructure enabling this research. We investigated the willingness to donate samples and linked clinical data for research purposes, and examined preferences regarding the receipt of research-derived information, including incidental findings.

**Methods:**

A structured, self-administered questionnaire was distributed electronically to a representative sample of the Israeli population. Participants were asked about their willingness to donate biosamples and linked data, and their opinions regarding receiving results including incidental findings. The data were analyzed by sociodemographic and health characteristics.

**Results:**

The survey included 1,607 respondents. 52% were willing to donate biosamples, and 40% were also willing to link them with their medical records and to receive incidental results (84.6% of those willing to donate). Individuals most willing to donate and receive research findings, were non-ultra-Orthodox Jewish, secular, male, aged 30 to 49 years with post-secondary/academic education and above-average income. Additionally, respondents characterized by better general health, being physically active, and absence of chronic or severe illness were more willing to donate biosamples. The main barriers to biosample donation were concerns about data leakage, privacy violations and lack of understanding of the donation purpose.

**Conclusion:**

Israel’s biosample donation landscape mirrors global patterns, with notable sectorial and demographic disparities. Higher socioeconomic status and health engagement increase willingness to donate, while privacy concerns and the perceived burden of continued involvement reduce participation.

## Background

Biospecimens are essential resources for modern medical and biological research with a potential to expand knowledge about genetic, behavioral, and environmental causes of diseases, support the development of new diagnostic methods and therapies, and improve medical care toward more personalized medicine. Biobanks are repositories that collect, process, store, and distribute human biospecimens and their associated data for research and clinical care. Initially small and project-specific, the improvements in biospecimen handling, storage and analysis technologies have led to the establishment of large-scale institutional, commercial, population-based and virtual repositories [1]. Modern biobanks now manage complex datasets, including genetic and proteomic information. At the same time regulatory and ethical considerations have developed to address issues such as informed consent, data privacy, ownership of biological materials, and the responsible return of research results to participants [2, 3]. Population-wide biobanks exist in many countries to gather phenotypic and genetic data on representative populations [4]. For example, the UK Biobank, holds over 500,00 biosamples of British residents and has made its data available to researchers worldwide, who are investigating a wide range of genetic and environmental factors underlying common diseases [5].

Following global trends, Israel has entered an era of large longitudinal studies in personalized medicine, particularly in the field of genetics, conducted mainly under the National Digital Health Project. Israeli society showcases remarkable ethnic, cultural, religious and genetic diversity. Among the country’s 10 million residents in 2025, 77% of the population are Jews, including Ashkenazi (descended from Central and Eastern Europe), Mizrahi and Sephardi (from the Middle East, North Africa, and the Mediterranean), Ethiopian Jews, and smaller groups like Cochin and Bene Israel, often with mixed heritage [6]. About 21% are Arabs, including Muslims (85% of all Israeli Arabs, and including Bedouins), Christians (9.0%), Druze (6%) and some smaller ethnic groups such as the Circassians, Armenian, and Samaritains. An additional 2.5% are neither Jewish nor Arab [7]. Therefore, there is great value in ensuring that biobanking initiatives and research efforts are designed to reflect this rich mosaic of communities, so that findings are representative, culturally sensitive, and applicable to the full spectrum of Israel’s diverse population.

Israel’s biobanking framework has evolved from early private initiatives in the 1990s to a more structured national effort. In 2012, a governmental forum decided to establish a national biobank for research [8]. MIDGAM - The Israeli National Biobank for Research was established by the Ministry of Health in 2014 as an infrastructure for the advancement of biomedical research and industry in Israel and for international collaboration. MIDGAM incorporates biobanks from several major tertiary medical centers across Israel and provides solutions at a national level to professional and ethical problems in the field of biobanking. The participating medical centers serve as collection sites, with secure, coded sample storage and structured access to a coded database for approved researchers from academia and industry across Israel and from abroad. Only coded samples are provided to researchers while access to associated identified medical data is restricted to authorized personnel from MIDGAM [8]. The process is overseen by the Ministry of Health and approved by its Supreme Independent Review Board. The biosamples are linked to clinical data in order to enable integrated research, allowing investigators to correlate biological findings with real-world clinical data, for example, identifying biomarkers associated with disease progression, treatment response, or long-term outcomes.

Since the founding of MIDGAM, additional health-maintenance organizations and institutional biobanks were established.

Biosample donation is a complex process shaped by individuals’ understanding of biobanking, their trust in governmental and research organizations, their views on the anticipated benefits, and their cultural or religious attitudes toward different kinds of biological materials [9, 10]. The need for human samples is steadily increasing, with requirements becoming increasingly specific over time. Furthermore, longitudinal studies require prolonged contact between the donors and the research team, with continuous transfer of data.

We conducted a survey to evaluate the willingness of potential donors to donate samples and linked data for research purposes. Additionally, we explored their preferences regarding the receipt of research-derived information, including incidental findings, particularly, genetic data. Finally, we aimed to identify the key motivating factors and concerns associated with such donations.

## Methods

### Study design, setting and participants

The study questionnaire was distributed by Kantar (Givatayim, Israel) between January and March 2020 using its established online panel of respondents. A targeted sampling approach was applied to recruit individuals aged 18–65 years and to approximate the demographic distribution of the Israeli population. Invitations were distributed via digital channels to panel members meeting predefined quota criteria (e.g., age, gender, and sector) until the required sample size was achieved. Responses were collected through a structured online questionnaire completed on participants’ personal devices (e.g., personal computers, tablets, or smartphones) via a secure web-based questionnaire platform. Subsequent data processing included quality control procedures and statistical weighting to further align the sample with national population benchmarks.

The study was approved by the Israeli Ministry of Health’s Supreme Independent Review Board (approval number MID-111–2019) and was conducted in accordance with the principles of the Declaration of Helsinki. The survey included a statement indicating that completion of the questionnaire constituted consent to participate. Participants were free to discontinue the survey at any time without any consequences.

### Study questionnaire

A structured, self-administered questionnaire was specifically developed for this study. An explanation was provided in the questionnaire that biological samples comprise tissue samples collected during an elective medical procedure that the donor is undergoing as part of a diagnostic work-up or treatment of their medical condition; blood samples collected during a medical procedure or through intentional (planned) collection; other samples such as saliva, urine, hair, feces, and various bodily fluids. The participants were than asked the following questions:

If you are formally approached on behalf of MIDGAM or a digital health initiative: (1) Would you agree to donate biological samples and linked information for future research purposes? (possible responses: Yes/No). (2) Would you agree to donate biological samples and linked information on an ongoing basis? (possible responses: Yes, whenever needed/Yes, at most once a year/Yes, at most once every three years/No). (3) If the samples you donate are used in future research, would you like to receive research findings about yourself? (possible responses: Yes, all information, including genetic information/Yes, only information with potential therapeutic relevance/No; if the information has critical clinical significance, I request that it be communicated directly to my treating physician/Under no circumstances, even if the information has critical clinical significance).

In addition, detailed demographic and personal information were collected including gender, age, education, income, place of residence, marital status, religion, level of religious observance, and ethnicity. Information on medical history (personal and family-related), comorbidities and history of hospitalizations was also collected.

### Study outcomes

The following outcomes were evaluated:

#### Concept assessment

The public’s readiness to participate in the project, alongside perceptions of the clarity and trustworthiness of the donation request. Motivating factors and perceived barriers to participation were also explored.

#### Target audience identification

The willingness of respondents to participate in MIDGAM and their interest in receiving feedback data were assessed. Respondents were characterized according to demographic variables (gender, age, education, income, residential area, marital status, religion, level of religiosity, and ethnicity) and medical history (personal and family history, comorbidities, and previous hospitalizations).

### Statistical analysis

The data were summarized using descriptive statistics. Study variables were summarized by frequency and percentages. Differences between the groups were tested for significance using the chi-square test for nominal variables and the Mann-Whitney test for ordinal variables. In cases where more than two levels were compared, each level was tested against all other levels combined. Statistical significance was set at *p* < 0.05.

## Results

### Characteristics of the study population

The survey included 1,607 Israeli men and women aged 18 to 65 years. The male to female ratio was 1.0, and 44.8% of respondents were 30–49 years old. Most respondents (72.0%) were non-ultra-Orthodox Jews, 9.0% were ultra-Orthodox Jews, and 19.0% were Arabs. Over half of respondents (56.7%) reported that they are secular, 68.7% were married or living with a partner, and 60.0% had post-secondary or academic education (Table 1).

**Table 1:** Socio-demographic characteristics of the study population

Characteristic		Study population N=1607 n (%)	Respondents willing to donate biosamples N=836 n (%)	P-value	Respondents willing to donate biosamples and interested in incidental findings N=643 n (%)	P-value
Sector	Non-ultra-Orthodox Jewish	1157 (72.0)	652 (78.0)	< 0.0001	511 (79.4)	< 0.0001
	Ultra-Orthodox Jewish	145 (9.0)	58 (6.9)	0.0032	41 (6.3)	0.0035
	Arab	305 (19.0)	126 (15.1)	< 0.0001	91 (14.2)	0.0001
Gender	Male	815 (50.7)	456 (54.6)	0.0016	366 (56.9)	0.0001
	Female	792 (49.3)	380 (45.4)		277 (43.1)	
Age	18–29	456 (28.4)	197 (23.6)	< 0.0001	142 (22.1)	< 0.0001
	30–49	720 (44.8)	380 (45.4)		289 (44.9)	
	50–65	429 (26.7)	259 (31.0)		212 (33.0)	
Education	≤ 8 years of school/primary school education	45 (2.8)	13 (1.6)	< 0.0001	10 (1.6)	< 0.0001
	12 years (high school education)	598 (37.2)	273 (32.7)		190 (29.6)	
	Post-secondary/academic education	964 (60.0)	550 (65.8)		443 (68.9)	
Income per houshold*	Below average	513 (31.9)	237 (28.3)	< 0.0001	174 (27.1)	< 0.0001
	Average	407 (25.3)	223 (26.7)		172 (26.8)	
	Above average	482 (30.0)	303 (36.2)		242 (37.6)	
	Refused to respond	204 (12.7)	74 (8.8)		55 (8.5)	
Religiosity	Secular	911 (56.7)	500 (59.8)	0.01	381 (59.3)	0.0914
	Traditional	376 (23.4)	181 (21.7)	0.0962	143 (22.3)	0.4162
	Religious	174 (10.8)	96 (11.5)	0.4235	77 (12.0)	0.2537
	Orthodox	146 (9.1)	58 (6.9)	0.0024	41 (6.4)	0.0028
Marital status	Married/living with a partner	1104 (68.7)	574 (68.7)	1	449 (69.8)	0.4226
	Bachelor	397 (24.7)	195 (23.3)	0.2017	141 (22.0)	0.0429
	Separated	106 (6.6)	67 (8.0)	0.0223	53 (8.2)	0.0375

*According to the Central Bureau of Statistics, in 2019, the average net income per household in Israel was ILS 16,559

### Willingness to donate among the general sample

Over half of the respondents (*n* = 836; 52%) were willing to donate biosamples and data for research purposes. Among these, 707 individuals (84.6%; 44% of the total sample) were also willing to link their biosamples with their medical records.

**Fig. 1:**
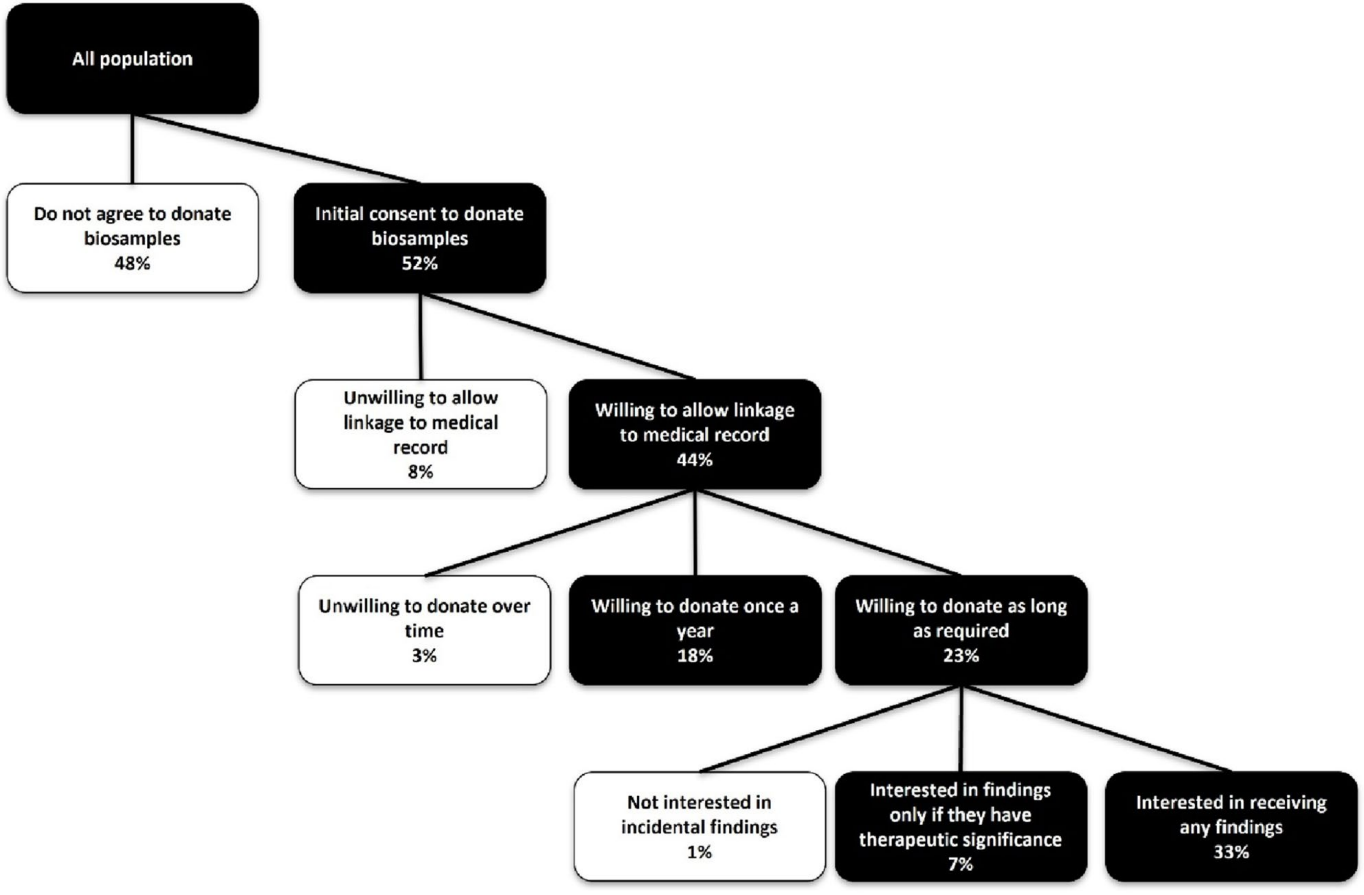
The rate of willingness to donate and to receive research findings among study participants (*N* = 1607)

Among those willing to donate biosamples with linkage to medical records, 659 (93.2%; 41% of the total sample) were willing to donate biosamples at least once a year. Specifically, 370 respondents (56.2%; 23% of the total sample) were willing to donate as needed, including multiple times, while 289 (43.9%; 18% of the total sample) preferred to donate once a year (Fig. 1).

A similar proportion of respondents to those willing to donate biosamples linked to medical records at least once a year (*n* = 643, 40% of the total sample) were interested in receiving information about incidental findings from their samples. Among them, 530 (82.4%; 33% of the total sample) wanted to be informed of any incidental findings, while 113 (17.6%; 18% of the total sample) were only interested in findings with therapeutic relevance (Fig. 1).

Among those interested in receiving information about incidental findings related to their samples (*n* = 819), 70% preferred to receive information via traditional mail or email. About 29% preferred communication through a genetic counsellor, and 22% through their health maintenance organization-affiliated treating physician (respondents could select more than one option).

### Sociodemographic and health characteristics associated with willingness to donate biosamples

As shown in Table 1, individuals most willing to donate biosamples and receive research findings, were non-ultra-Orthodox Jewish, secular, male, aged 30 to 49 years with post-secondary/academic education and above-average income.

Analysis by population sectors showed that over half of non-Orthodox Jewish respondents (652/1157, 56.4%) were willing to donate biosamples in comparison to 41.3% (126/305) of the Arab respondents and 40% (58/145) of ultra-Orthodox Jewish respondents. A significant association was found between sectorial group and willingness to donate biosamples at least once a year, with a statistically significantly greater proportion of non-ultra-Orthodox Jewish respondents (683/1157, 59%) willing to donate biosamples at least once a year, compared to Arab (140/305, 46%) and ultra-Orthodox (60/85, 41%) respondents (χ²[2, *N* = 1607] = 28.7, *p* < 0.001). A significant association was also found between sectorial group and interest in incidental findings, with 44.2% (511/1157) of non-ultra-Orthodox Jewish respondents willing to donate and interested in incidental findings compared to 29.8% (91/305) of Arab respondents and 28.3% (41/145) of ultra-Orthodox Jewish respondents (χ²[2, *N* = 1607] = 29.7, *p* < 0.001).

Table 2 summarizes the relationship between participants’ health characteristics and their willingness to donate biosamples and receive incidental findings. Respondents who showed greater willingness to donate biosamples reported being physically active, non-smokers, with no chronic disease, history of severe illness or a family history of genetic diseases. Additionally, respondents who were overweight (body mass index [BMI] > 25 kg/m^2^) and those who reported previous hospitalizations were also more willing to donate biosamples. Similar results were observed among respondents who were willing to donate samples and receive incidental findings; however, in this group there was no difference between smokers and non-smokers nor between those with a family history of genetic disease and those without. No statistically significant difference in the willingness to donate and receive incidental findings was observed between respondents who had had genetic counselling and those who did not.

**Table 2:** Distribution of health and lifestyle characteristics among survey respondents by willingness to donate biosamples and receive research findings

Characteristic		Study population N=1607 n (%)	Respondents willing to donate biosamples N=836 n (%)	*P*-value	Respondents willing to donate biosamples and interested in incidental findings N=643 n (%)	*P*-value
Physical activity	Physically active	865 (53.8%)	520 (62.2%)	< 0.0001	399 (62.1%)	< 0.0001
	Not physically active	742 (46.2)	316 (37.8%)		244 (37.9%)	
BMI	BMI > 25	834 (51.9%)	462 (55.2%)	0.0058	354 (55.1%)	0.0395
	BMI ≤ 25	773 (48.1%)	374 (44.8%)		289 (44.9%)	
Smoking status	Smoker or past smoker	516 (32.1%)	293 (35.0%)	0.0101	217 (33.7%)	0.2617
	Non-smoker	1091 (67.9%)	543 (65.0%)		426 (66.3%)	
Chronic illness	No	1290 (80.3%)	623 (74.5%)	< 0.0001	471 (73.2%)	< 0.0001
	Yes	317 (19.7%)	213 (25.5%)		172 (26.8%)	
History of severe illness	No	1488 (92.6%)	753 (90.1%)	0.0001	577 (89.8%)	0.0009
	Yes	119 (7.4%)	83 (9.9%)		66 (10.2%)	
Previous hospitalizations	No	858 (53.4%)	395 (47.2%)	< 0.0001	301 (46.8%)	< 0.0001
	Yes	749 (46.6%)	441 (52.8%)		342 (53.2%)	
Family history of genetic disease	No	1523 (94.8%)	783 (93.6%)	0.0483	606 (94.2%)	0.6295
	Yes	84 (5.2%)	53 (6.4%)		37 (5.8%)	
Genetic counseling	No	1159 (72.1%)	589 (70.5%)	0.1345	458 (71.2%)	0.5966
	Yes	448 (27.9%)	247 (29.5%)		185 (28.8%)	

### Barriers to donating and receiving research findings

**Fig. 2:**
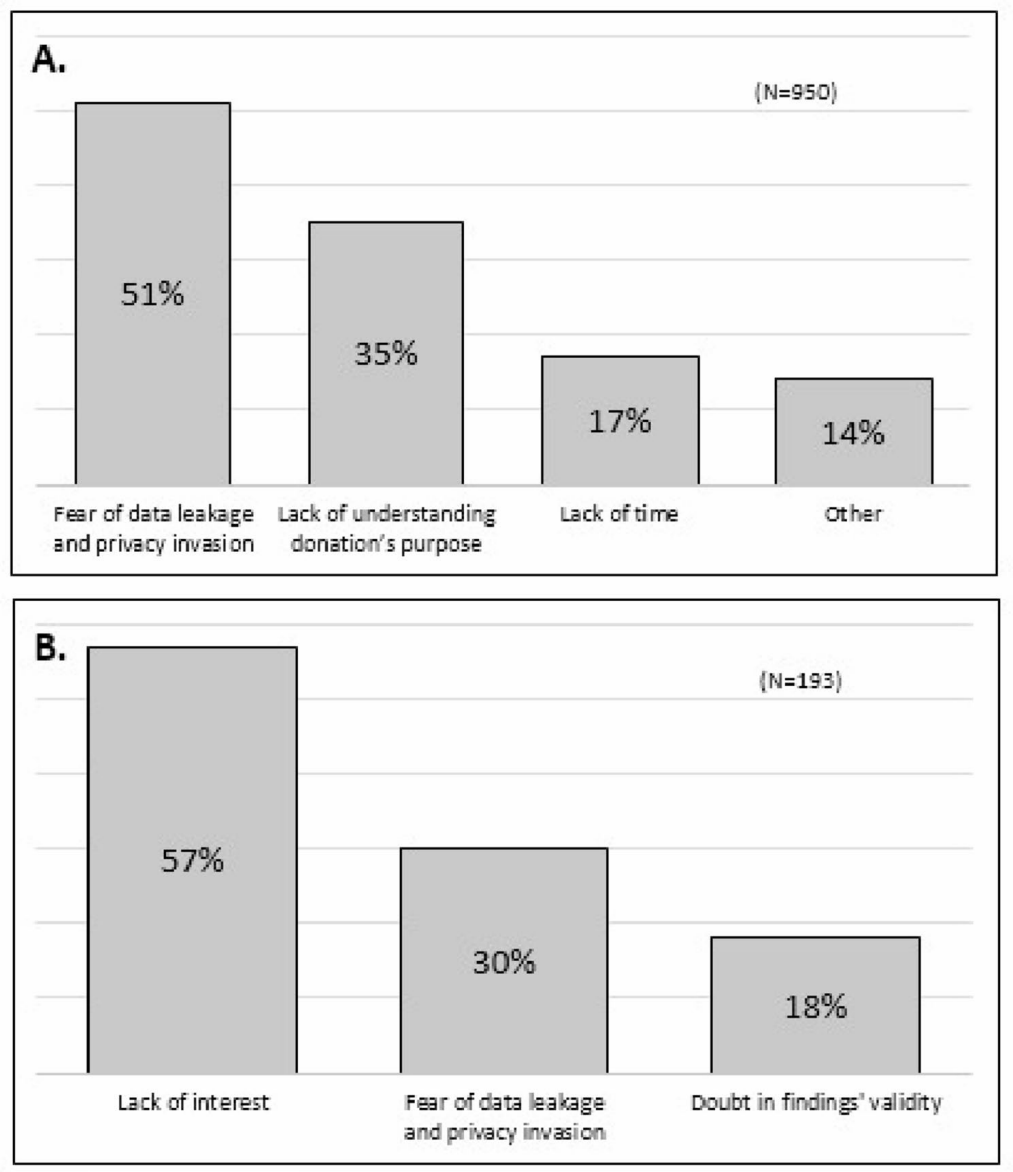
(**A**) The main reasons for refusal to donate biosamples over time (% of the sample). (**B**) Reasons for the reluctance to receive incidental findings (% of the sample)

Among the 950 respondents who were unwilling to donate a biosample or receive findings, the main barriers were concerns about data protection and privacy (51%), and a lack of understanding of the donation’s purpose (35%) (Fig. 2A). Among non-ultra-Orthodox Jewish respondents, the most common concern was data protection and privacy (56%), whereas the proportions of Arab and ultra-Orthodox respondents who were concerned about this issue was lower (42% and 38%, respectively). Among respondents who would not want to receive incidental finding, 57% cited lack of interest, 30% were concerned about data leakage and invasion of privacy and 18% expressed doubt in the validity of findings (Fig. 2B).

We examined whether various explanations from treating physicians could influence the willingness to donate biosamples, such as emphasizing population representation, curing specific diseases, or contributing to equitable healthcare resource distribution. However, 91% of respondents in this group indicated that they would be unpersuaded by explanations by treating physicians, indicating their views are unlikely to change.

## Discussion

This study provides a comprehensive assessment of the willingness of Israeli adults to donate biosamples and linked clinical data for research. Our analysis showed that just over half (52%) of respondents would be willing to donate biosamples. Among those willing to donate, most (84.6%) also agreed to approve linkage to their medical records, and over 90% were willing to donate biosamples on a recurring basis, either annually or as needed.

The proportion of respondents who were willing to donate biosamples was higher than that reported for Latvia (40.7%) [11] and Poland (47.6%) [12], but lower than the rate reported in studies conducted in Jordan (64%) [13], China (where 66% would be extremely willing to moderately willing to donate) [14], the United Kingdom (75%), Finland (83%) [15] and Sweden (86%) [16]. Very low willingness to donate (less than 10%) was found in low- and middle-income countries of the Arab region (Egypt, Jordan, Morocco, and Sudan) [17]. A pan-European study has found a strong association between a country’s level of engagement in biobanking (having heard or talked about biobanks) and the proportion of individuals willing to donate biosamples [9].

We found that older respondents as well as those with higher education and income were more likely to donate biosamples. Similarly, Ahram et al. [18] found a significant correlation between higher income and higher education and the likeliness to donate biospecimens for biobanking. In contrast to our findings, they found that older individuals (above 60 years) had a more negative view of biobanking; however, our study population only included respondents up to 65 years of age.

In this study, most respondents willing to donate biosamples (84.6%) also agreed to link them with their electronic medical records. In a study conducted in the United Kingdom, 68% of respondents said they would agree to have their medical records and lifestyle information linked to the donated biosample but deidentified, whereas 82% were willing to have their deidentified lifestyle information linked to their biosamples [19]. However, data protection and privacy concerns emerged as chief barriers across all groups, especially among non-ultra-Orthodox Jews (56%) compared to Arabs (42%) and ultra-Orthodox (38%). Concerns about data protection and privacy, often shaped by prior interactions with healthcare systems, were expressed in surveys on the willingness to donate biosamples across the globe [14, 15, 17, 19–24]. This underscores the global importance of trust and privacy in biobanking recruitment.

Cultural and social and differences associated with different types of spirituality or religiosity may affect the willingness to donate biosamples. Lower levels of support for human biosample donations were observed among minority ethnic groups in the United Kingdom [19], African-Americans [21, 25–27] and Asian-American women [22] in the United States. In our study, analysis by population sectors revealed that willingness to donate was highest among non-ultra-Orthodox Jewish respondents, whereas lower willingness to donate was observed among ultra-Orthodox and Arab populations. Ultra-Orthodox Jews follow strict religious laws. Several studies have identified an inverse relationship between belief in God or religiosity level and the willingness to share information with a biobank. The attitudes of Malay-Muslims in Singapore toward donating biospecimens and participating in biobanking have been reported to be negatively influenced by perceived religious beliefs [28]. In a study conducted in the United Kingdom, respondents who wanted to be asked to donate human biological samples were significantly more likely to be either not religious or only moderately so [19]. In a study conducted among Polish medical students, both religious and nonreligious students supported the idea of biobanking of human biological material and were willing to donate for research purposes, however, nonreligious students felt more positive toward biobanking, supported the idea of establishing biobanks in Poland more often, and were more eager to donate most types of tissues and to participate in biobank research [29]. In a study conducted in Jordan, 61.2% of participants responded that religious permission of donating biospecimens for the purpose of scientific research would positively influence their decision to donate biosamples [18]. Therefore, securing the endorsement of religious leaders could help address and alleviate objections to donation among individuals with stronger religious beliefs.

Notably, 40% of the total population expressed interest in receiving incidental findings from research, with a majority preferring full disclosure of results, while a smaller subset expressed interest only in findings with therapeutic relevance. This preference may reflect a growing public expectation for transparency, autonomy, and access to personal health information. Increased awareness of personalized medicine and genetic testing, along with greater engagement in healthcare decision-making, may contribute to individuals’ desire to receive all available information, including findings not directly related to the initial purpose of sample collection. Additionally, participants may perceive potential personal or clinical utility in such information, even when its significance is uncertain. In other studies, returning research results was found to be an incentive to drive public donation for biobanking [18, 27, 30, 31], especially in studies requiring multiple donations over time [31]. In light of this finding, biobanks may consider adopting policies that support tiered or dynamic consent models, allowing participants to specify their preferences regarding the return of incidental findings (e.g., all findings, only clinically actionable findings, or no return). Policies could also incorporate clear frameworks for determining which results are eligible for return, prioritizing those with established clinical validity and utility. Notably, while returning research results offers several benefits, including demonstrating respect for participants, fostering their trust, enhancing their understanding, and increasing their engagement with research efforts, this practice also presents challenges, such as requiring substantial financial and logistical resources, the potential risk of inaccurately conveying findings, and the possibility that sharing research data may cause psychological distress or social difficulties without providing clear clinical benefit [32, 33].

Overall, participants characterized by better general health, being physically active, and absence of chronic or severe illness demonstrated significantly greater willingness to donate biosamples. Other studies have shown that individuals with cancer and other diseases were more willing to donate biosamples compared to the general public [34–37]. Differences in study context, population characteristics, or the fact that our study focused on linking data with medical records or donations over time may have contributed to divergence from prior findings, suggesting that willingness to donate biosamples cannot be assumed to be uniformly higher among individuals with existing health conditions.

Willingness to donate biological samples to biobanks has been consistently linked to broader attitudinal and psychological constructs, particularly trust, values, and perceptions of risk [12, 38]. Evidence suggests that individuals who are willing to donate are more likely to exhibit higher levels of trust in healthcare professionals and scientific institutions, which has been identified as one of the strongest predictors of participation [12, 16, 38]. Given that negative or burdensome healthcare experiences may erode such trust, it is plausible that individuals with prior healthcare encounters may exhibit greater caution toward participation in biobanking or research activities. Such individuals may also be more cautious about the implications of receiving incidental findings, including uncertain or anxiety-provoking results. Beyond trust, value-based orientations, such as altruism, personal development, and contribution to scientific progress, have been positively associated with donation willingness, whereas classical personality traits appear to play a more limited or inconsistent role [12, 38, 39]. Therefore, willingness to participate in biobanking seems to be shaped by a multidimensional interplay of psychological, social, and institutional factors, rather than by a single underlying construct [40].

The willingness to donate biosamples over an unlimited period appeared to be a limiting factor, even after providing an initial consent to donate and link the biosamples to clinical data. Among those who would be willing to donate biosamples, 46% agreed to donate as needed, 43% preferred annual donations, and 11% declined ongoing participation, suggesting long-term, open-ended donation may deter continued involvement.

While educational interventions, such as informed explanations from treating physicians, have been shown to shift attitudes in some populations [19], our findings indicate minimal impact on individuals initially inclined to decline. This suggests deeply held concerns that generalized messaging alone may be insufficient, especially among reticent subgroups. Correspondingly, international studies show that face-to-face engagement outperforms passive informational material in enhancing biobank participation.

### Strength and limitations

The strength of our study is the involvement of a representative sample of the Israeli population and the examination of multiple factors. However, several limitations related to the use of an online survey should be acknowledged. First, reliance on an online panel may introduce selection bias, as participation is limited to individuals with internet access and a willingness to engage in digital surveys, potentially underrepresenting older adults, individuals of lower socioeconomic status, or those with lower digital literacy. Second, online surveys are subject to self-selection bias, whereby individuals with stronger opinions or greater interest in the topic may be more likely to participate. Conversely, individuals may be reluctant to explicitly state their views objectively and might provide socially acceptable answers. Third, compared with telephone or face-to-face methods, online surveys lack the opportunity for clarification or probing, which may increase the risk of misinterpretation of questions. Finally, although quality control measures were applied, online data collection may be more susceptible to issues such as inattentive responding or survey satisficing. These factors may limit the generalizability of the findings and should be considered when interpreting the results.

### Policy implications

The findings of this study offer practical and actionable insights for strengthening Israel’s national biobanking infrastructure. Specifically, they can inform MIDGAM’s public-engagement strategies by identifying the populations most willing to participate, clarifying the central barriers that deter donation, and highlighting differences across demographic and cultural groups. By leveraging these insights, MIDGAM can develop targeted communication approaches, enhance transparency, address privacy-related concerns more directly, and tailor its outreach to Israel’s diverse communities. In turn, these efforts will improve public trust, promote equitable access to biobanking initiatives, and support the long-term sustainability and national impact of Israel’s biobank.

## Conclusions

Israel’s biosample donation landscape is shaped by similar demographic, cultural, and attitudinal patterns seen globally with significant sectorial, demographic, and behavioral disparities in attitudes toward donation and return of results. Socioeconomic advantage and health engagement predict willingness to participate, while privacy concerns and the perceived burden of ongoing commitment limit participation.

## Data Availability

The datasets generated and analyzed during the current study are available from the corresponding author upon reasonable request.

## References

[1] De Souza YG, Greenspan JS. Biobanking past, present and future: responsibilities and benefits. AIDS. 2013;27(3):303–12. 10.1097/QAD.0b013e32835c1244. 23135167 10.1097/QAD.0b013e32835c1244 PMC3894636

[2] Amoakoh-Coleman M, Vieira D, Abugri J. Ethical considerations for biobanking and use of genomics data in Africa: a narrative review. BMC Med Ethics. 2023;24(1):108. 10.1186/s12910-023-00985-y. 38053109 10.1186/s12910-023-00985-y PMC10699036

[3] Husedzinovic A, Ose D, Schickhardt C, Fröhling S, Winkler EC. Stakeholders’ perspectives on biobank-based genomic research: systematic review of the literature. Eur J Hum Genet. 2015;23(12):1607–14. 10.1038/ejhg.2015.27. 25735479 10.1038/ejhg.2015.27 PMC4795193

[4] Lieb W, Strathmann EA, Röder C, Jacobs G, Gaede KI, Richter G, et al. Population-based biobanking. Genes (Basel). 2024. 10.3390/genes15010066. 38254956 10.3390/genes15010066 PMC10815030

[5] Manolio TA. UK Biobank debuts as a powerful resource for genomic research. Nat Med. 2018;24(12):1792–4. 10.1038/s41591-018-0276-3. 30510254 10.1038/s41591-018-0276-3

[6] Shalev SA, Zlotogora J, Shalata A, Levy-Lahad E. Medical genetics in Israel’s diverse population. Lancet. 2017;389(10088):2453–5. 10.1016/S0140-6736(17)30875-9. 28495108 10.1016/S0140-6736(17)30875-9

[7] Central Bureau of Statistics. Israel’s Independence Day 2025. https://www.cbs.gov.il/en/mediarelease/Pages/2025/Israel-Independence-Day-2025.aspx (2025). Accessed 5 June 2025.

[8] Siegal G. Genomic databases and biobanks in Israel. J Law Med Ethics. 2015;43(4):766–75. 10.1111/jlme.12318. 26711416 10.1111/jlme.12318

[9] Gaskell G, Gottweis H, Starkbaum J, Gerber MM, Broerse J, Gottweis U, et al. Publics and biobanks: Pan-European diversity and the challenge of responsible innovation. Eur J Hum Genet. 2013;21(1):14–20. 10.1038/ejhg.2012.104. 22669414 10.1038/ejhg.2012.104 PMC3522201

[10] Domaradzki J, Pawlikowski J. Public attitudes toward biobanking of human biological material for research purposes: a literature review. Int J Environ Res Public Health. 2019. 10.3390/ijerph16122209. 31234457 10.3390/ijerph16122209 PMC6617000

[11] Mezinska S, Kaleja J, Mileiko I, Santare D, Rovite V, Tzivian L. Public awareness of and attitudes towards research biobanks in Latvia. BMC Med Ethics. 2020;21(1):65. 10.1186/s12910-020-00506-1. 32736554 10.1186/s12910-020-00506-1 PMC7393882

[12] Pawlikowski J, Wiechetek M, Majchrowska A. Associations between the willingness to donate samples to biobanks and selected psychological variables. Int J Environ Res Public Health. 2022;19(5):2552. 35270246 10.3390/ijerph19052552 PMC8910049

[13] Ahram M, Othman A, Shahrouri M. Public perception towards biobanking in Jordan. Biopreserv Biobank. 2012;10(4):361–5. 10.1089/bio.2012.0010. 24849885 10.1089/bio.2012.0010

[14] Huang M, Yu L, Wang X, Li K, Wang J, Cheng X, et al. Public awareness, attitudes, and motivation toward biobanks: a survey of China. BMC Med Ethics. 2025;26(1):2. 10.1186/s12910-025-01163-y. 39799302 10.1186/s12910-025-01163-y PMC11724581

[15] Tupasela A, Sihvo S, Snell K, Jallinoja P, Aro AR, Hemminki E. Attitudes towards biomedical use of tissue sample collections, consent, and biobanks among Finns. Scand J Public Health. 2010;38(1):46–52. 10.1177/1403494809353824. 19906772 10.1177/1403494809353824

[16] Kettis-Lindblad A, Ring L, Viberth E, Hansson MG. Genetic research and donation of tissue samples to biobanks. What do potential sample donors in the Swedish general public think? Eur J Public Health. 2006;16(4):433–40. 10.1093/eurpub/cki198. 16207726 10.1093/eurpub/cki198

[17] Ahram M, Abdelgawad F, ElHafeez SA, Abdelhafiz AS, Ibrahim ME, Elgamri A, et al. Perceptions, attitudes, and willingness of the public in low- and middle-income countries of the Arab region to participate in biobank research. BMC Med Ethics. 2022;23(1):122. 10.1186/s12910-022-00855-z. 36457067 10.1186/s12910-022-00855-z PMC9713115

[18] Ahram M, Othman A, Shahrouri M, Mustafa E. Factors influencing public participation in biobanking. Eur J Hum Genet. 2014;22(4):445–51. 10.1038/ejhg.2013.174. 23921537 10.1038/ejhg.2013.174 PMC3953902

[19] Lewis C, Clotworthy M, Hilton S, Magee C, Robertson MJ, Stubbins LJ, et al. Public views on the donation and use of human biological samples in biomedical research: a mixed methods study. BMJ Open. 2013. 10.1136/bmjopen-2013-003056. 23929915 10.1136/bmjopen-2013-003056 PMC3740256

[20] Beskow LM, Dean E. Informed consent for biorepositories: assessing prospective participants’ understanding and opinions. Cancer Epidemiol Biomarkers Prev. 2008;17(6):1440–51. 10.1158/1055-9965.Epi-08-0086. 18559560 10.1158/1055-9965.EPI-08-0086

[21] Drake BF, Boyd D, Carter K, Gehlert S, Thompson VS. Barriers and strategies to participation in tissue research among African-American men. J Cancer Educ. 2017;32(1):51–8. 10.1007/s13187-015-0905-1. 26341221 10.1007/s13187-015-0905-1 PMC4779426

[22] Lee CI, Bassett LW, Leng M, Maliski SL, Pezeshki BB, Wells CJ, et al. Patients’ willingness to participate in a breast cancer biobank at screening mammogram. Breast Cancer Res Treat. 2012;136(3):899–906. 10.1007/s10549-012-2324-x. 23129174 10.1007/s10549-012-2324-x PMC3676182

[23] Ma Y, Dai H, Wang L, Zhu L, Zou H, Kong X. Consent for use of clinical leftover biosample: a survey among Chinese patients and the general public. PLoS One. 2012;7(4):e36050. 10.1371/journal.pone.0036050. 22558323 10.1371/journal.pone.0036050 PMC3338618

[24] Gao Z, Huang Y, Yao F, Zhou Z. Public awareness and attitudes toward biobank and sample donation: a regional Chinese survey. Front Public Health. 2022;10:1025775. 10.3389/fpubh.2022.1025775. 36504979 10.3389/fpubh.2022.1025775 PMC9727410

[25] Moorman PG, Skinner CS, Evans JP, Newman B, Sorenson JR, Calingaert B, et al. Racial differences in enrolment in a cancer genetics registry. Cancer Epidemiol Biomarkers Prev. 2004;13(8):1349–54. 15298957

[26] Wang SS, Fridinger F, Sheedy KM, Khoury MJ. Public attitudes regarding the donation and storage of blood specimens for genetic research. Community Genet. 2001;4(1):18–26. 10.1159/000051152. 11493749 10.1159/000051152

[27] Hoeyer K, Olofsson BO, Mjörndal T, Lynöe N. Informed consent and biobanks: a population-based study of attitudes towards tissue donation for genetic research. Scand J Public Health. 2004;32(3):224–9. 10.1080/14034940310019506. 15204184 10.1080/14034940310019506

[28] Wong ML, Chia KS, Wee S, Chia SE, Lee J, Koh WP, et al. Concerns over participation in genetic research among Malay-Muslims, Chinese and Indians in Singapore: a focus group study. Public Health Genomics. 2004;7(1):44–54. 10.1159/000080303. 10.1159/000080303 15475670

[29] Domaradzki J, Walkowiak D. When biobanks meet religion: association between religiosity and attitudes of Polish medical students toward biobanking of human biological material for research purposes. J Relig Health. 2024;63(2):1178–213. 10.1007/s10943-023-01932-2. 37847446 10.1007/s10943-023-01932-2 PMC10965646

[30] Beskow LM, Friedman JY, Hardy NC, Lin L, Weinfurt KP. Simplifying informed consent for biorepositories: stakeholder perspectives. Genet Med. 2010;12(9):567–72. 10.1097/GIM.0b013e3181ead64d. 20697289 10.1097/GIM.0b013e3181ead64d PMC3250643

[31] Murphy J, Scott J, Kaufman D, Geller G, LeRoy L, Hudson K. Public expectations for return of results from large-cohort genetic research. Am J Bioeth. 2008;8(11):36–43. 10.1080/15265160802513093. 19061108 10.1080/15265160802513093 PMC2682364

[32] Bookman EB, Langehorne AA, Eckfeldt JH, Glass KC, Jarvik GP, Klag M, et al. Reporting genetic results in research studies: summary and recommendations of an NHLBI working group. Am J Med Genet A. 2006;140(10):1033–40. 10.1002/ajmg.a.31195. 16575896 10.1002/ajmg.a.31195 PMC2556074

[33] Shalowitz DI, Miller FG. Communicating the results of clinical research to participants: attitudes, practices, and future directions. PLoS Med. 2008;5(5):e91. 10.1371/journal.pmed.0050091. 18479180 10.1371/journal.pmed.0050091 PMC2375946

[34] Ahram M, Zaza R, Ibayyan L, Dahbour S, Bahou Y, El-Omar A, et al. Towards establishing a multiple sclerosis biobank in Jordan. Int J Neurosci. 2014;124(11):812–7. 10.3109/00207454.2014.886204. 24456262 10.3109/00207454.2014.886204

[35] Pentz RD, Billot L, Wendler D. Research on stored biological samples: views of African American and White American cancer patients. Am J Med Genet A. 2006;140(7):733–9. 10.1002/ajmg.a.31154. 16523508 10.1002/ajmg.a.31154

[36] Drake BF, Brown K, McGowan LD, Haslag-Minoff J, Kaphingst K. Secondary consent to biospecimen use in a prostate cancer biorepository. BMC Res Notes. 2016;9:346. 10.1186/s13104-016-2159-3. 27431491 10.1186/s13104-016-2159-3 PMC4949745

[37] Domaradzki J, Czekajewska J, Walkowiak D. Attitudes of oncology patients’ towards biospecimen donation for biobank research. BMC Cancer. 2024;24(1):390. 10.1186/s12885-024-12145-5. 38539134 10.1186/s12885-024-12145-5 PMC10967165

[38] Sedlár M, Grežo M. Willingness to participate in biobanking: the roles of Big Five personality traits and interpersonal trusting beliefs. Pers Individ Dif. 2022;197:111770. 10.1016/j.paid.2022.111770.

[39] Grežo M, Sedlár M. Public’s awareness of biobanks and willingness to participate in biobanking: the moderating role of social value orientation. J Community Genet. 2023;14(3):275–85. 10.1007/s12687-023-00634-2. 36662375 10.1007/s12687-023-00634-2 PMC10272000

[40] Domaradzki J, Walkowiak MP, Walkowiak D. Cluster donation: how future healthcare professionals bound certain types of tissues and biomedical research and how it affects their willingness to donate. Healthcare. 2023;11(19):2636. 37830675 10.3390/healthcare11192636 PMC10572418

